# Isorhamnetin inhibited migration and invasion via suppression of Akt/ERK-mediated epithelial-to-mesenchymal transition (EMT) in A549 human non-small-cell lung cancer cells

**DOI:** 10.1042/BSR20190159

**Published:** 2019-09-20

**Authors:** Wei Luo, Qingbin Liu, Nan Jiang, Mingquan Li, Li Shi

**Affiliations:** Affiliated Hospital of Changchun University of Chinese Medicine, Changchun 130021, China

**Keywords:** Akt/ERK1/2, Epithelial-mesenchymal transition (EMT), Isorhamnetin, Lung cancer, Metastasis

## Abstract

In the present study, we investigated the potential effects of Isorhamnetin on the growth and metastasis of A549 human lung cancer cells, as well as the underlying mechanism. Treatment with Isorhamnetin exhibited a dose- and time-dependent inhibition on A549 cell proliferation. Furthermore, the cell adhesion and Transwell assay showed that treatment with Isorhamnetin (2.5, 5, and 10 μM) for 48 h resulted in a significant inhibition effect on cell adhesion, invasion and migration of A549 cells, depending on concentration, which was associated with the suppression of matrix metalloproteinase (MMP)-2 and MMP-9 activity and protein expression. Moreover, Isorhamnetin effectively suppressed the expressions of epithelial-to-mesenchymal transition (EMT) markers, as evidenced by the down-regulation of N-cadherin, vimentin and snail, as well as up-regulation of E-cadherin protein expression. Additionally, these inhibitions were mediated by interrupting AKT/ERK1/2 signaling pathways. Taken together, the results of the current study demonstrated that Isorhamnetin may become a good anti-metastastic agent against lung cancer A549 cell line by the suppression of EMT via interrupting Akt/ERK1/2 signaling pathway.

## Introduction

Lung cancer is the most prominent causes of cancer-related mortality worldwide and the overall five years survival is approximately 15% [1,2]. Of the lung cancer cases diagnosed, nearly 80% are non-small-cell lung cancer (NSCLC), which has a poor prognosis and limited therapeutic options, due to metastasis [3,4]. Cancer cell metastasis is the leading cause of cancer mortality and is a complex multistep process involving invasion and migration [5,6]. In many cases, local invasion and/or metastasis from primary sites to distant organs are responsible for the poor prognosis and treatment failure in patients with advanced NSCLC [7]. Despite the improvement of the early diagnosis and the development of platinum-based chemotherapy during the past few decades, the prognosis and the survival rate of patients with NSCLC remains unsatisfactory. Consequently, it is urgent to develop metastasis preventive strategies and to find new, safe and efficient preventive agents for the treatment of lung cancer

**Figure 1:**
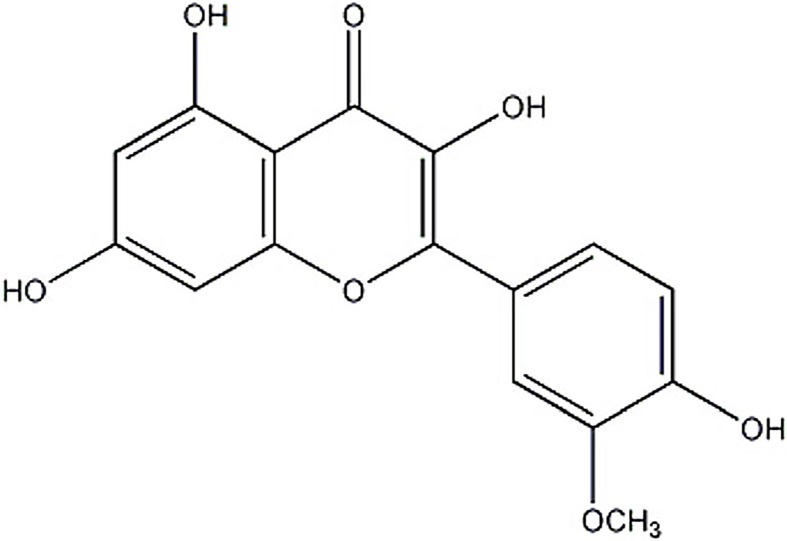
Chemical structure of Isorhamnetin

Traditional Chinese medicine is a treasure trove gifted by nature to discover novel compounds to prevent cancer metastasis [8]. Isorhamnetin (3′-methoxy-3,4′,5,7-tetrahydroxyflavone; Figure 1), a flavonol aglycone, is rich in fruits, vegetables, and tea, as well as traditional medicine, such as *Hippophae rhamnoides* (L.) [9],*Vernonia anthelmintica* (L.) [10], and *Astragalus membranaceus* (Fisch.) [11]. Shenqi Fuzheng Injection also contained this ingredient and possessed growth inhibitory effect toward lung cancer cell line [12,13]. Several lines of evidence indicated that this compound was used to treat cardiovascular disease, rheumatism, and hemorrhage [14,15]. Of note, its anticancer effects have also been reported including lung cancers. Ruan et al. [16] showed that inhibition of autophagy may be a useful strategy for enhancing the chemotherapeutic effect of Isorhamnetin on lung cancer cells. It has been proved that Isorhamnetin has a synergetic effect to enhance the anticancer activity and apoptosis induction of cisplatin and carboplatin in NSCLC A549 cell line [17]. Despite this background, there is lack of information about anti-metastatic potential of isorhamnetin on NSCLC. In view of this condition, the aim of the present study was to evaluate the anti-metastasis activities of Isorhamnetin on A549 cell cells and to illustrate the specific mechanism involved in these effects.

## Materials and methods

### Reagents

Isorhamnetin with a purity of up to 98% was purchased from Sigma–Aldrich (St. Louis, MO, U.S.A.) and was initially dissolved in dimethyl sulfoxide (DMSO) (Sigma) at a stock solution of 100 mM and stored at −20°C. For treatment of cells, Isorhamnetin was diluted with medium to the appropriate concentrations prior to use, and the final concentration of DMSO was <0.01%.

### Cell culture and 3-(4,5-dimethylthiazol-2-yl)-2,5-diphenyltetrazolium bromide assay

Human lung adenocarcinoma cell line A549 was obtained from Maoxin Biotechnology Co. Ltd. (Xi’an, China) and were maintained in Dulbecco’s Modified Eagle Medium (DMEM, Gibco, Grand Island, NY, U.S.A.) supplemented with 10% fetal bovine serum (FBS; BioWest, Nuaillé, France), 100 U/ml penicillin and 100 U/ml streptomycin in a 37°C incubator with a 5% (v/v) CO_2_ humidified atmosphere for 24 h. Then, cells were seeded into 96-well plate and treated with either 1% serum DMEM as a control or various concentrations of Isorhamnetin (2.5, 5, and 10 μM) for 24, 48, and 72 h, respectively. After treatment, the cells were incubated for 4 h in the presence of 20 μl of 3-(4,5-dimethylthiazol-2-yl)-2,5-diphenyltetrazolium bromide assay (MTT, 5 mg/ml, Sigma–Aldrich, St. Louis, MO, U.S.A.) at 37°C, followed by the addition of 150 μl (DMSO, Sigma–Aldrich) to dissolve the formazan crystals. After thorough mixing, the absorbance of the wells at 570 nm was measured using a Model 550 microplate reader (Bio-Rad Laboratories, Inc., Hercules, CA, U.S.A.). Cell viability was calculated as a percentage of MTT reduction relative to untreated cells (designed as 100%). At least three independent experiments were performed.

### Colony formation assay

A549 cells suspended in 5 ml of DMEM was plated in triplicate into a six-well plate (1 × 10^4^ cells/well) and incubated with different dose of Isorhamnetin (2.5, 5, and 10 μM) for 48 h. The fresh medium containing tested drug or not was changed every three days. After treatment of 12 days, the supernatant was discarded and then the resulting colonies were fixed with 5% paraformaldehyde at 4°C for 15 min followed by staining with 0.05% crystal violet (Sigma–Aldrich) solution for 30 min. Finally, the stained colonies images were captured under an inverted microscope (Nikon, Tokyo, Japan). Assays were carried out in triplicate on three independent experiments.

### Cell adhesion assay

A cell adhesion experiment was measured using the MTT assay as described for the cell viability assay. Briefly, The A549 cells (5 × 10^4^ cells/well) pre-incubated with different concentrations of isorhamnetin for indicated time at 37°C was inoculated into the 96-well plate coated with Matrigel (BD Biosciences, Franklin Lakes, NJ, U.S.A.) and allowed to adhere for 1 h at 37°C. Unattached cells were removed by gentle washing with PBS and attached cells in each well were stained with 0.1% crystal violet for 10 min, and then subjected to taking photograph under a Leica DMI 400B microscope. Subsequently, crystal violet was lysed with 30% acetic acid, and optical density (OD) of each well was measured at 570 nm with a reference 405 nm on a microplate reader (Rayto, RT-6000). The adhesion rate was calculated from the OD values of triplicate experiments.

### Cell invasion and migration assays

*In vitro* invasion and migration assays were performed in transwell chambers (Corning Costar, MA, U.S.A.) with 8-μm pore size polycarbonate filter according to the methods reported by Repesh [18], with minor modifications. For migration assay, after treatment, A549 cells suspended in 100 μl serum-free DMEM were placed in the upper transwell chamber at cell density of 1 × 10^5^ cells/well and medium supplemented with 10% FBS was added into the lower chamber as chemoattractant. Following incubation at 37°C for 24 h, the cells on the upper surface of the filter were completely removed with a cotton swab and cells invaded to the underside of the membrane were stained with 0.2% Crystal violet in 2% ethanol (15 min). These cells that migrated into the lower surface of the filter were then quantified by counting at least five random fields and photographed under a DMI 8 inverted microscope (Leica Microsystems, Wetzlar, Germany) (×20). For migration assay, each upper side of filter was coated overnight with 100 μl of Matrigel (2 mg/ml, diluted in cold serum-free DMEM without chemoattractant; Corning, NY, U.S.A.) to form a thin homogeneous film on the top of the filter and experimental processes were operated in the same condition as *in vitro* invasion assay. Each experiment was carried out in triplicate, and data were expressed as migration and invasion rate to represent the average cell number.

### Gelatin zymography

The enzymatic activities of matrix metalloproteinase (MMP)-2 and MMP-9 in the conditioned medium of A549 cells were assayed using gelatin zymography on 10% sodium dodecyl sulfate/polyacrylamide gel electrophoresis (SDS/PAGE) containing 2.56 mg/ml gelatin after A549 cells were treated with various concentrations of Isorhamnetin (2.5, 5, and 10 μM) for 48 h. Following electrophoresis, the gel was washed with distilled water in a solution containing 2.5% (v/v) Triton X-100 at 37°C, and then transferred to a reaction buffer (50 mmol/l Tris-HCl, 10 mmol/ CaCl_2_ and 0. 02% Brij-35, pH 8.0) at 37°C with gentle agitation for 15 h. Finally, the gels were stained with 0.5% (w/v) Coomassie Blue R-250 (Beijing Solarbio Science & Technology Co., Ltd., Beijing, China) for 30 min at 25°C and de-stained with 10% acetic acid, 50% methanol, and 40% of water at 25°C for 2 h. The relative MMP-2 and MMP-9 activities were compared with control group and quantified by densitometer measurement using a digital imaging analysis system (GeneGenius, Syngene, U.K.).

### Plasmids and cell transfections

Plasmids and cell transfections were performed as described previously [19]. cDNAs encoding constitutively active ERK or AKT were all designed and synthesized by Shanghai Gene Pharma Company (Shanghai, China) and subcloned into pcDNA3.1 vector (Invitrogen, Carlsbad, CA, U.S.A.) to yield pcDNA3.1(+)-ERK or pcDNA3.1(+)-AKT. A549 cells were seeded into six-well plates until approximately 60% confluence before transfection. The next day, all plasmids and empty vector (control) were transfected into target cells using Lipofectamine 2000 (Invitrogen, Carlsbad, CA, U.S.A.) according to the manufacturer’s protocol. After 48 h transfection, cells were harvested and lysed to confirm the success overexpression and the resulting proteins were used for Western blot analysis.

### Western blot analysis

Following treatment, cells were harvested with ice-cold PBS and whole-cell lysates were prepared by lysing with an extraction buffer [20 mM Tris-Cl (pH 7.5), 200 mM NaCl, 5 mM ethylenediaminetetra acetic acid, 2 μg/ml leupeptin, 1% Nonidet P-40, 100 μg/ml phenylmethylsulfonyl fluoride, and 0.1 mM sodium orthovanadate (Roche Molecular Diagnostics, Pleasanton, CA, U.S.A.)]. Protein concentration was determined with a Bio-Rad protein assay kit (Bio-Rad, Hercules, CA, U.S.A.) according to the manufacturer’s protocol. For Western blot analysis, 50 mg total protein was separated using 10% SDS/PAGE and then transferred to nitrocellulose membranes (Millipore, Bedford, MA, U.S.A.). After blocking with 5% skimmed milk solution at 37°C for 1 h, the membranes were subjected to incubation with the appropriate primary antibody against MMP-2, MMP-9, E-cadherin, N-cadherin, Vimentin, slug, snail, phospho-Akt, Akt, phospho-ERR1/2, ERK1/2, phospho-JNK1/2, JNK1/2, phospho-p38, p-38, or β-actin overnight at 4°C, and then with secondary horseradish peroxidase-coupled antibodies at room temperature for 45 min. Following three washes with ice-cold PBS, blots were developed using an enhanced chemiluminescence detection kit (GE Healthcare Life Sciences, Shanghai, China) and the intensities of protein expression were then quantitated by an Image Lab v.4.62 (Bio-Rad Laboratories, Inc.). Equal loading of samples was confirmed by probing the membranes with a β-actin antibody as the internal control. Primary antibodies were purchased from Santa Cruz Biotechnology Inc. (Santa Cruz, CA, U.S.A.) and secondary horseradish peroxidase-coupled antibodies were purchased from Cell Signaling Technologies, (Boston, U.S.A.).

### Statistical analysis

Each experiment was carried out at least three times and expressed as the mean ± standard deviation (S.D.). One-way analysis of variance (ANOVA) tests w ereused for multiple comparisons and Student’s *t* test was used to evaluate the differences between two groups. A value of *P*<0.05 was considered significant.

## Results

### Isorhamnetin inhibits the growth of A549 cells

**Figure 2:**
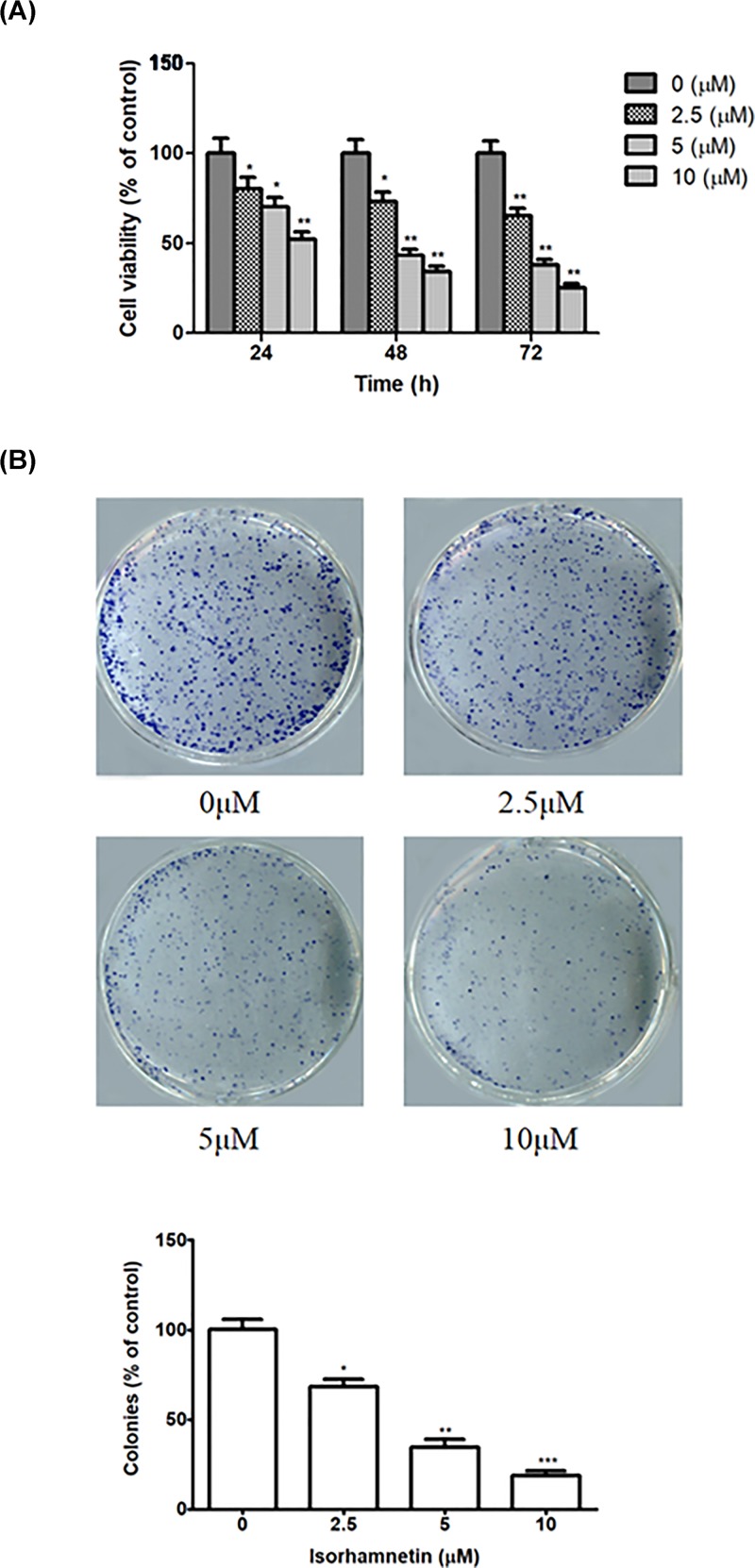
Isorhamnetin inhibits the growth of A549 cells (**A**) Effect of Isorhamnetin (2.5, 5, and 10 μM) on the proliferation of A549 lung cancer cells at 24, 48, and 72 h. (**B**) Effect of Isorhamnetin (2.5, 5, and 10 μM) on colony formation of A549 cells. Bars represent mean ± S.D. (*n*=3). *P<0.05, **P<0.01, and ***P<0.001 were considered to indicate a statistically significant difference compared with the control.

After treatment with Isorhamnetin (2.5, 5, and 10 μM) for 24, 48, and 72 h, MTT assay showed that the cell viability decreased with rising dose and extended time. Comparing with that of control, the viability of A549 cells was significantly affected at three doses and three incubation period (Figure 2A). At the period of 48 h, tumor growth inhibition effect is more evident than that in 24 h, and is so close to maximum inhibitory effect occurred after 72 h. Based on the data from the MTT assay, we selected three different concentrations of Isorhamnetin (2.5, 5, and 10 μM) and the period of 48 h for further investigation. Then we conducted colony formation test to support the MTT result. As expected, treatment with Isorhamnetin (2.5, 5, and 10 μM) significantly suppressed the colony formation ability of A549 cells in a dose-dependent manner, as evidenced by decreased size and number of colonies in treated cells than the control (Figure 2B).

### Isorhamnetin inhibits the adhesion, migration, and invasion of A549 cells

**Figure 3:**
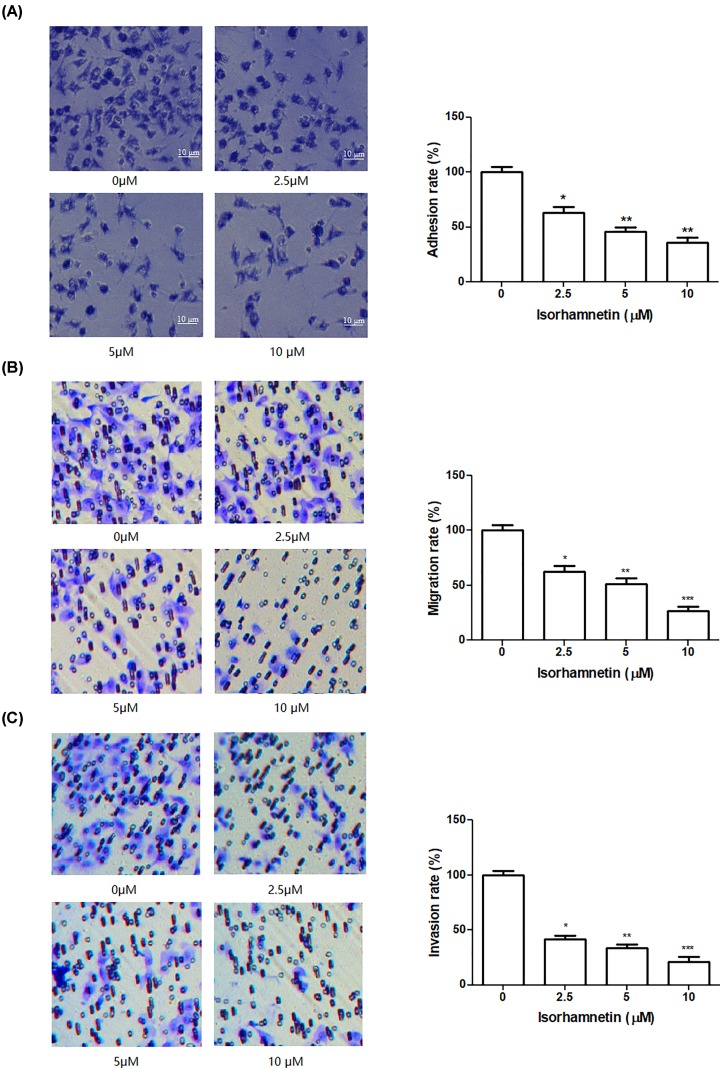
Isorhamnetin inhibits the growth of A549 cells (**A**) Effects of Isorhamnetin (2.5, 5, and 10 μM) on the adhesion of A549 lung cancer cells. (**B**) Effects of Isorhamnetin (2.5, 5, and 10 μM) on the migration of A549 lung cancer cells. (**C**) Effects of Isorhamnetin (2.5, 5, and 10 μM) on the invasion of A549 lung cancer cells. Bars represent mean ± S.D. (*n*=3). ^*^P<0.05, ^**^P<0.01, and ^***^P<0.001 were considered to indicate a statistically significant difference compared with the control.

Cell-matrix adhesion, cell invasion, and cell motility are important for cancer cell metastasis. To examine the anti-metastasis behavior of Isorhamnetin against A549 cells, *in vitro* adhesion, migration, and invasion assay was conducted one by one in cells following Isorhamnetin treatment (2.5, 5, and 10 μM) for 48 h. Figure 3A illustrates that Isorhamnetin inhibited the adhesion of the A549 cells to Matrigel in a concentration-dependent manner compared with the untreated control cells, and the cell adhesion rates of the A549 cells were 63.27% (*P*<0.05), 45.77% (*P*<0.05), and 36.17% (*P*<0.01) relative to control group, respectively. Meanwhile, Transwell migration and invasion assay results showed that Isorhamnetin significantly inhibited the migration and invasion of A549 cancer cells with an increasing concentration of Isorhamnetin. The addition of Isorhamnetin led to an obvious decrease of the migration of A549 cancer cells by 62.67% at 2.5 μM (*P*<0.05), 51.33% at 5 μM (*P*<0.01), and 26.67% at 10 μM (*P*<0.001) of Isorhamnetin, compared with the control (Figure 3B). The invasive activities of A549 cells were significantly reduced to 41.56% (*P*<0.05), 33.41% (*P*<0.01), and 21.20% (*P*<0.01) by treatment of 2.5, 5, and 10 μM of Isorhamnetin, respectively, compared with untreated group (Figure 3C). These results suggested that Isorhamnetin exerted an anti-adhesive, anti-migrative, and anti-invasive ability toward A549 cells at moderate concentrations.

### Isorhamnetin inhibits the activation of MMP-2 and MMP-9 in A549 cells

**Figure 4:**
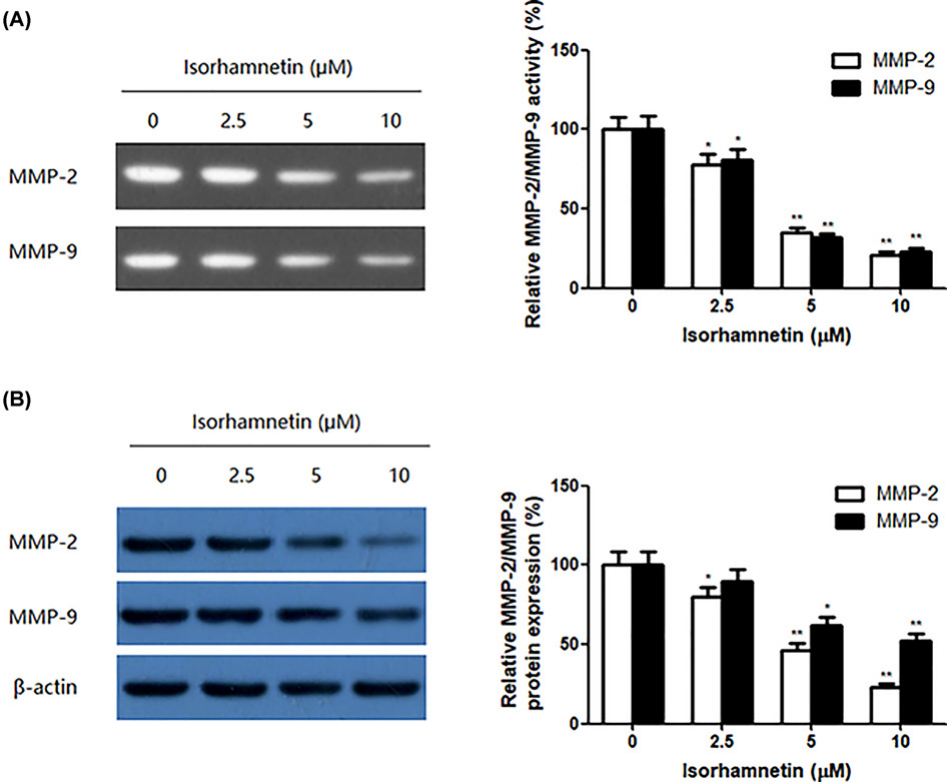
Isorhamnetin inhibits the activation of MMP-2 and MMP-9 in A549 cells (**A**) Effects of Isorhamnetin (2.5, 5, and 10 μM) on the activities of MMP-2 and MMP-9 in culture media of A549 lung cancer cells (**B**) Effects of Isorhamnetin (2.5, 5, and 10 μM) on the protein expression of MMP-2 and MMP-9 from whole-cell lysates of A549 lung cancer cells. Bars represent mean ± S.D. (*n*=3). ^*^P<0.05 and ^**^P<0.01 were considered to indicate a statistically significant difference compared with the control.

To examine whether the metastasis inhibitory effect of Isorhamnetin resulted from the suppression of MMPs, the activity of MMP-2/MMP-9 in culture media of A549 cells and protein expression of MMP-2/MMP-9 from whole-cell lysates of A549 cells were measured by gelatin zymography and Western blotting assay, respectively. As seen in Figure 4A, gelatinase activity of MMP-2 and MMP-9 were suppressed in a dose-dependent manner in the serum-free medium of A549 cells by treatment of Isorhamnetin (2.5, 5, and 10 μM), with a maximum inhibition of 21.10% for MMP-2 and 23.02% for MMP-9 at 10 μM of Isorhamnetin, compared with the control (*P*<0.01). Similarly, the MMP-2 and MMP-9 protein expression from whole-cell lysates of A549 cells were reduced to 80.01% (*P*<0.05), 46.01% (*P*<0.01), and 23.01% (*P*<0.01) for MMP-2, and 89.69% (*P*>0.05), 62.05% (*P*<0.01), and 52.06% (*P*<0.01) for MMP-9 by the addition of 2.5, 5, and 10 μM of Isorhamnetin, respectively, compare with untreated group (Figure 4B). This change was in accordance with those of the MMP-2 activity assay. These data indicate that the anti-metastatic effect of Isorhamnetin in A549 cells is related to inhibition of the enzyme activity of MMP-2 and MMP-9.

### Isorhamnetin reduced the expressions of cell invasion-related and EMT-related molecules in A549 Cells

**Figure 5:**
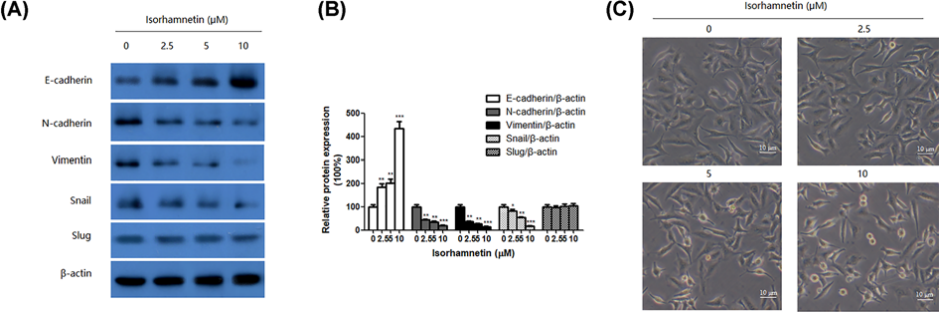
Isorhamnetin inhibits the activation of MMP-2 and MMP-9 in A549 cells (**A**) Effects of Isorhamnetin (0, 2.5, 5, and 10 μM) on the protein expression of E-cadherin, N-cadherin, vimentin, snail, and slug of A549 lung cancer cells. (**B**) Quantitative result of E-cadherin, N-cadherin, vimentin, snail, and slug proteins expression of A549 lung cancer cells treated with Isorhamnetin (0, 2.5, 5, and 10 μM). (**C**) Effects of Isorhamnetin (2.5, 5, and 10 μM) on the cellular morphology of A549 lung cancer cells. Bars represent mean ± S.D. (*n*=3). ^*^P<0.05, ^**^P<0.01, and ^***^P<0.001 were considered to indicate a statistically significant difference compared with the control.

EMT is a fundamental cell biological process that is often involved in embryogenesis and diseases, such as cancer invasion and metastasis [20]. So we examined the EMT marker proteins such as E-cadherin, N-cadherin, and vimentin, as well as corresponding transcriptional factor (snail and slug) in A549 cells in the presence and absence of Isorhamnetin (2.5, 5, and 10 μM). The result from Western blotting (Figure 5A,B) showed that treatment with Isorhamnetin (2.5, 5, and 10 μM) significantly enhanced epithelial-like cell marker E-cadherin expression in A549 cells by 185.63% (*P*<0.01), 201.57% (*P*<0.01), and 438.56% (*P*<0.001), respectively. On the contrary, the addition of Isorhamnetin (2.5, 5, and 10 μM) remarkably decreased the protein expression of mesenchymal-like cell marker by 43.33% (*P*<0.01), 35.23% (*P*<0.01), and 20.21% (*P*<0.001) for N-cadherin, and 36.16% (*P*<0.01), 26.23% (*P*<0.01), and 15.01% (*P*<0.001) for vimentin, respectively. Meanwhile, transcription factor, snail, but not slug, was dramatically decreased in response to isorhamnetin treatment. These results indicated that Isorhamnetin could interfere with EMT process to prevent the metastasis of A549 lung cancer cells.

In line with this change, untreated control cells displayed a spindle-shaped cellular morphology. In contrast, cells cultured with Isorhamnetin (2.5, 5, and 10 μM) exhibited polygonal or cobblestone-like shape (Figure 5C).

### Isorhamnetin inhibits phosphorylation of ERK and Akt

**Figure 6:**
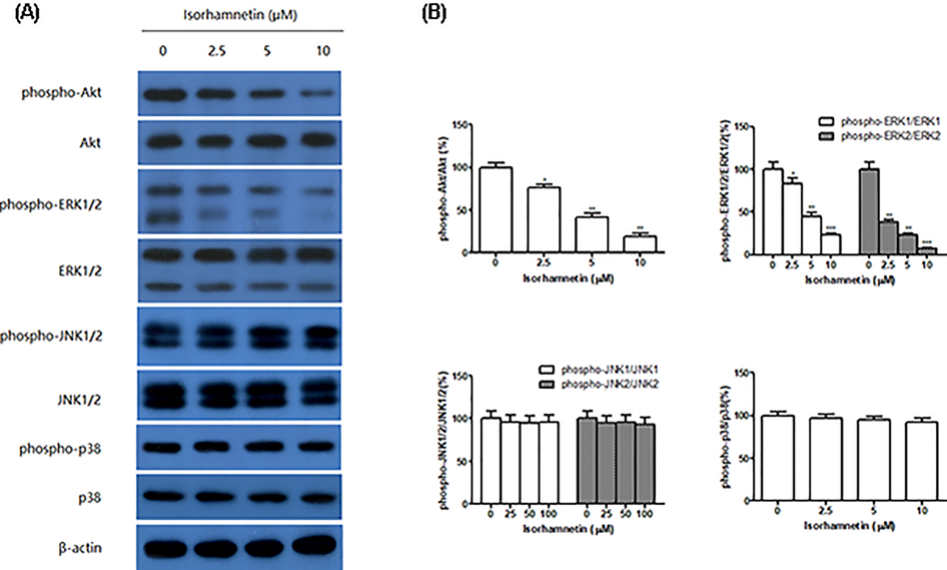
Isorhamnetin inhibits phosphorylation of ERK and Akt in A549 cells (**A**) Effects of Isorhamnetin (2.5, 5, and 10 μM) on the protein expression of phosphor-Akt, Akt, phosphor-ERK1/2, ERK1/2, phosphor-JNK1/2, JNK1/2, phosphor-p38, and p38 of A549 lung cancer cells. (**B**) Quantitative result of phosphor-Akt, Akt, phosphor-ERK1/2, ERK1/2, phosphor-JNK1/2, JNK1/2, phosphor-p38, and p38 proteins expression of A549 lung cancer cells. Bars represent mean ± S.D. (*n*=3). ^*^P<0.05, ^**^P<0.01, and ^***^P<0.001 were considered to indicate a statistically significant difference compared with the control.

In order to further investigate the underlying mechanism, the effects of Isorhamnetin (2.5, 5, and 10 μM) on the protein expression levels of phosphorylated MAPK family members (JNK1/2, ERK1/2, and p38 MAPK) and Akt were examined using Western blot analysis in A549 cells. As seen in Figure 6A,B, activation of ERK1/2 and Akt in A549 cells were both significantly inhibited by Isorhamnetin (2.5, 5, and 10 μM), along with the decreasing phosphorylation of ERK1/2 and Akt. However, no any change of phospho-JNK1/2 and phospho-p38 expression was present in A549 cells in response to Isorhamnetin (2.5, 5, and 10 μM).

**Figure 7:**
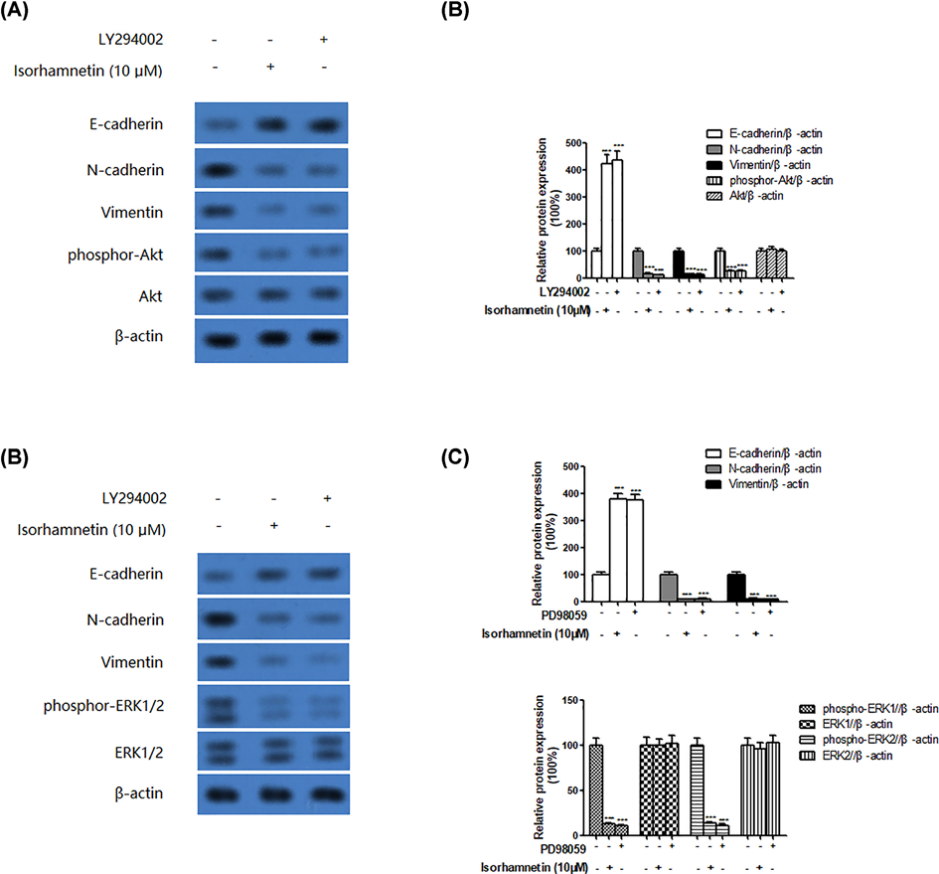
Effect of P13K/AKT or ERK inhibitor on EMT in A549 cells (**A**) Effects of PI3K/Akt inhibitor (LY294002) or Isorhamnetin (10 μM) on the protein expression of E-cadherin, N-cadherin, vimentin, phospho-Akt, and Akt of A549 lung cancer cells. (**B**) Quantitative result of E-cadherin, N-cadherin, vimentin, phospho-Akt, and Akt proteins expression of A549 lung cancer cells treated with LY294002 or Isorhamnetin (10 μM). (**C**) Effects of ERK inhibitor (PD98059) or Isorhamnetin (10 μM) on the protein expression of E-cadherin, N-cadherin, vimentin, phospho-ERK1/2, and ERK1/2 of A549 lung cancer cells. (**D**) Quantitative result of E-cadherin, N-cadherin, vimentin, phospho-ERK1/2, and ERK1/2 proteins expression of A549 lung cancer cells treated with PD98059 or Isorhamnetin (10 μM). Bars represent mean ± S.D. (*n*=3). ^*^P<0.05, ^**^P<0.01, and ^***^P<0.001 were considered to indicate a statistically significant difference compared with the control.

In line with these results, the addition of PI3K/Akt inhibitor (LY294002) or ERK inhibitor (PD98059) 30 min before treatment of A549 cells with Isorhamnetin (10 μM) resulted in the induction of EMT, as evidenced by a up-regulation of E-cadherin and down-regulation of N-cadherin and vimentin (Figure 7A–D). Meanwhile, the decreasing phosphorylations of ERK1/2 and Akt were both observed in A549 cells treated with Isorhamnetin, LY294002 or PD98059 alone, but the total ERK1/2 and Akt protein kept unchanged.

**Figure 8:**
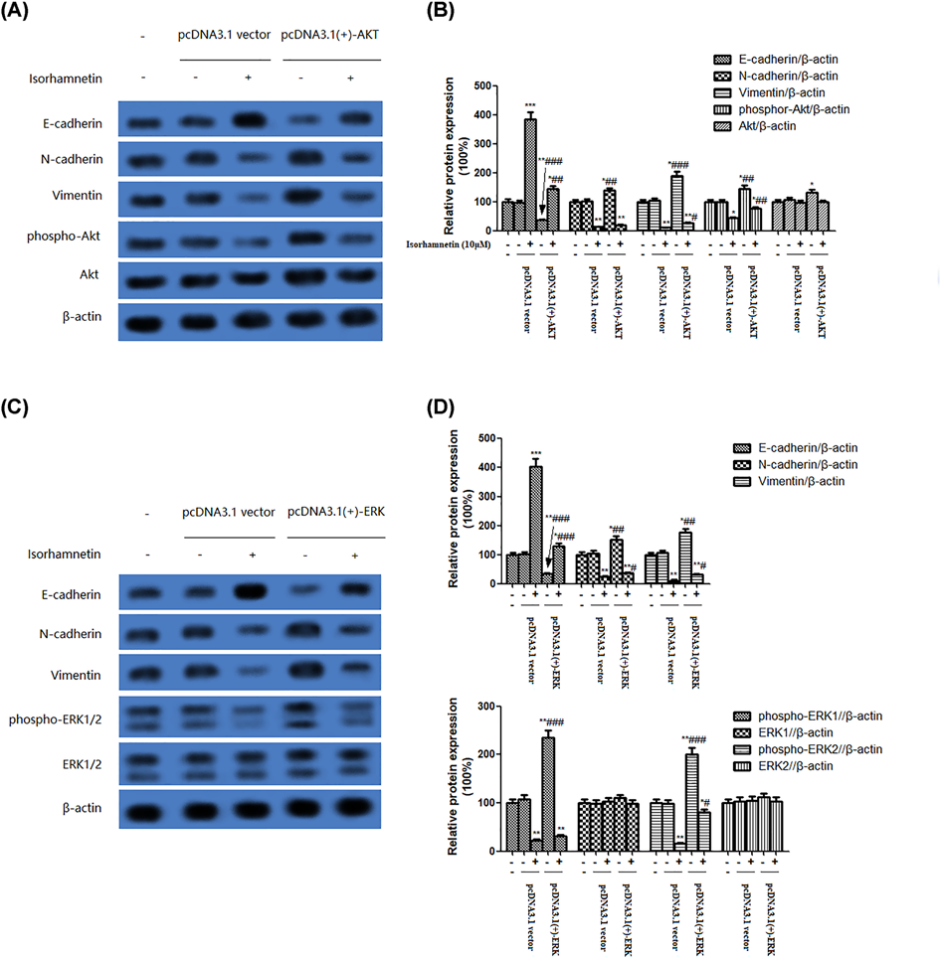
Effect of P13K/AKT or ERK overexpression on EMT in A549 cells (**A**) Effects of overexpression of PI3K/Akt on the protein expression of E-cadherin, N-cadherin, vimentin, phospho-Akt, and Akt of A549 lung cancer cells. (**B**) Quantitative result of E-cadherin, N-cadherin, vimentin, phospho-Akt, and Akt proteins expression of A549 lung cancer cells transfected with pcDNA3.1(+)-Akt or pcDNA3.1 vector. (**C**) Effects of overexpression of ERK on the protein expression of E-cadherin, N-cadherin, vimentin, phospho-ERK1/2, and ERK1/2 of A549 lung cancer cells. (**D**) Quantitative result of E-cadherin, N-cadherin, vimentin, phospho-ERK1/2, and ERK1/2 proteins expression of A549 lung cancer cells transfected with pcDNA3.1(+)-ERK or pcDNA3.1 vector. Bars represent mean ± S.D. (*n*=3). *P<0.05, **P<0.01, and ***P<0.001 were considered to indicate a statistically significant difference compared with the normal control. ^#^*P*<0.05, ^##^*P*<0.01, and ^###^*P*<0.001 were considered to indicate a statistically significant difference compared with the Isorhamnetin (10 μM)-treated control.

To evaluate in depth the role of the PI3K/Akt/ERK pathway in the induction of EMT of A549 cells, we further examined the effect of overexpression of Akt or ERK on the protein expression of E-cadherin, N-cadherin, vimentin, phospho-Akt, Akt or phospho-ERK1/2, and ERK1/2. As shown in Figure 8A–D, A549 cells transfected with Akt plasmids showed a visible decrease of E-cadherin protein expression and concurrent increase of N-cadherin, vimentin, and phospho-Akt protein expression, as well as a slight increase of Akt. The same tendency was observed in A549 cells-transfected ERK plasmids. Whereas, these changes in A549 cells transfected with Akt or ERK plasmids were reversed by Isorhamnetin (10 μM) to approach the level in A549 cells only treated with 10 μM of Isorhamnetin. Taken together, these observing strongly indicate that Isorhamnetin inhibits EMT via inactivation of PI3K/Akt/ERK signaling pathway, thus leading to the inhibition of metastasis of A549 lung cancer cells.

## Discussion and conclusions

Lung cancer invasion and metastasis are the primary factor for the failure of cancer treatment and for the poor prognosis of advanced NSCLC, which is responsible for its high mortality rates [21,22]. Thus, discovering new anti-metastatic agents is crucial for lung cancer treatment. Recently, a substantial amount of evidence has shown that many natural-product-based drugs got more attention by scientists due to their beneficial effects for various diseases, especially for tumor metastasis. Isorhamnetin, is an effective ingredient from traditional Chinese medicinal herb or Shenqi Fuzheng Injection, which has been commonly used to treat lung cancer in clinic [23]. In the present study, we examine the potential anti-metastasis effects of Isorhamnetin on A549 cells and the underlying mechanism involved. MTT and colony formation assay clearly showed that Isorhamnetin (2.5, 5, and 10 μM) can significantly inhibit the cell growth of A549 cells at different incubation period, especially at 48 h. To further examine the potential anti-metastasis effects of Isorhamnetin (2.5, 5, and 10 μM), adhesion, invasion, and migration assays were performed on A549 cells. A remarkable declined ability of cell-matrix adhesion, migration and invasion was observed in A549 cells following Isorhamnetin (2.5, 5, and 10 μM) treatment for 48 h and exhibited a dose-response association, suggesting Isorhamnetin can potently inhibit tumor cell adhesion, invasion, and metastasis of A549 cells.

Tumor invasion and metastasis require degradation of ECM, which enable cancer cells into the blood or lymphatic system and spread to other tissues or organs at distance [24]. In this process, the MMPs, particularly MMP-2 and MMP-9, are responsible for ECM degradation, predominantly controlling tumor growth, invasion, and tumor-induced angiogenesis [25,26]. More specifically, increased expression of MMPs is associated with an increased potential for metastasis [27]. However, no report is available on the inhibition of Isorhamnetin on MMPs in lung cancer cells. We subsequently investigated the effect of Isorhamnetin on the enzyme activity and protein expressions of invasion-related molecules MMP-2 and MMP-9 by gelatin zymography and Western blotting, respectively. Both assays demonstrated that Isorhamnetin (2.5, 5, and 10 μM) was able to significantly inhibit the activity and protein expression of MMP-2 and MMP-9, again exhibiting a dose-response tendency. This reduced protein expression and secretion of MMP-2 and MMP-9 may account for the inhibitory effects of Isorhamnetin on the invasion and migration of A549 cells.

Emerging evidence also indicates EMT is considered as one of the most critical steps in cancer malignancy, metastasis, and recurrence [28,29]. During EMT progression, cancer cells obtain mesenchymal characteristics such as increase of vimentin and N-cadherin and lose epithelial properties like decrease of E-cadherin [30]. The incidence of EMT in tumor cells is always indicative of the enhancement of tumor metastasis [31]. Thus, inhibiting or reversing EMT progression may benefit for preventing and treating cancer metastasis. In the present study, Isorhamnetin (2.5, 5, and 10 μM) significantly inhibited EMT progression by decreasing the protein expressions of N-cadherin, vimentin, and snail, while increasing that of E-cadherin, suggesting that Isorhamnetin led to EMT of A549 cells.

Several studies have indicated that EMT is regulated by oncogenic kinase signaling pathways, including PI3K/Akt and MAPK family members (JNK1/2, ERK1/2, and p38), and they are often up-regulated in cancer initiation and invasion. To investigate whether Isorhamnetin inhibits activation of PI3K/Akt and MAPK signaling pathways, the phosphorylated status of JNK1/2, ERK1/2, p38 MAPK, and Akt protein was detected in A549 cells treated with various concentrations of Isorhamnetin (2.5, 5, and 10 μM) for 48 h. As anticipated, activation of Akt and ERK1/2 was significantly inhibited by Isorhamnetin at three doses, as shown by decreasing the phosphorylation of Akt and ERK1/2 with increasing concentration of Isorhamnetin. In contrast, Isorhamnetin did not affect phospho-JNK1/2 and phospho-p38 activity. This strongly indicates that Isorhamnetin selectively inhibits the Akt and ERK1/2 signaling pathway, but not of JNK1/2 and p38 MAPK.

To further investigate the role of Akt and ERK1/2 signaling pathway in the regulation of EMT, PI3K/Akt inhibitor (LY294002) or ERK inhibitor (PD98059) was added into A549 cells 30 min before treatment with Isorhamnetin (10 μM) for 48 h. The addition of LY294002 or PD98059 resulted in the inhibition of phospho-Akt or phospho-ERK1/2, which were combined with down-regulation of N-cadherin and vimentin, and up-regulation of E-cadherin in A549 cells, thus leading to the suppression of EMT. Simultaneously, transfection with Akt or ERK plasmids induced a decrease of E-cadherin protein expression, but an increase of N-cadherin and vimentin protein expression in A549 cells, together with an increased phosphorylation of Akt or ERK1/2. Collectively, the data of the present study implied that inhibition of EMT via inactivation of Akt/ERK1/2 signaling pathway by Isorhamnetin treatment may contribute to block the progression of the metastasis of A549 cancer cells. Therefore, Cal Isorhamnetin might become a useful antitumorigenic compound for lung cancer treatment and/or prevention in the future.

## Abbreviations

ANOVA: one-way analysis of variance
DMEM: Dulbecco’s Modified Eagle Medium
DMSO: dimethyl sulfoxide
EMT: epithelial-to-mesenchymal transition
ERK: extracellular signal-regulated kinase
FBS: fetal bovine serum
JNK: c-jun N-terminal kinase
MAPK: microtubule associated protein kinase
MMP: matrix metalloproteinase
MTT: 3-(4,5-dimethylthiazol-2-yl)-2,5-diphenyltetrazolium bromide
NSCLC: non-small-cell lung cancer
OD: optical density
S.D.: standard deviation
SDS/PAGE: sodium dodecyl sulfate/polyacrylamide gel electrophoresis
