# Expression of Concern: Isorhamnetin inhibited migration and invasion via suppression of Akt/ERK-mediated epithelial-to-mesenchymal transition (EMT) in A549 human non-small cell lung cancer cells

**DOI:** 10.1042/BSR-20190159_EOC

**Published:** 2020-06-30

**Keywords:** Isorhamnetin, Lung cancer, Metastasis, Epithelial-mesenchymal transition (EMT), Akt/ERK1/2

The Editorial Office has been made aware of potential issues surrounding the scientific validity of this paper, hence has issued an expression of concern to notify readers whilst the Editorial Office investigates.

