# Correction: Isorhamnetin inhibited migration and invasion via suppression of Akt/ERK-mediated epithelial-to-mesenchymal transition (EMT) in A549 human non-small cell lung cancer cells

**DOI:** 10.1042/BSR-20190159_COR

**Published:** 2020-08-07

**Keywords:** Isorhamnetin, Lung cancer, Metastasis, Epithelial-mesenchymal transition (EMT), Akt/ERK1/2

This Correction follows an Expression of Concern relating to this article previously published by Portland Press.

The authors of the original paper “Isorhamnetin inhibited migration and invasion via suppression of Akt/ERK-mediated epithelial-to-mesenchymal transition (EMT) in A549 human non-small cell lung cancer cells” (*Biosci Rep* (2019) **39**(9), DOI: 10.1042/BSR20190159) would like to replace Figures 3 and 5 with a new set of images, due to an unintentional mistake when preparing the submission. In an attempt to adjust the images in Figure 3B,C to be consistent with Figure 3A-C had become discontinuous and blurry in appearance. The wrong image was uploaded in the final revision before publication for Figure 5, instead of the one that was originally submitted upon the second revision of the manuscript (this can be verified via the submission system records). The corrections made in this erratum will not alter the conclusion of the results. The authors apologize for any inconvenience or misunderstanding that these errors may have caused. The corrected Figures 3 and 5 are updated as shown in this correction article.

**Figure 3:**
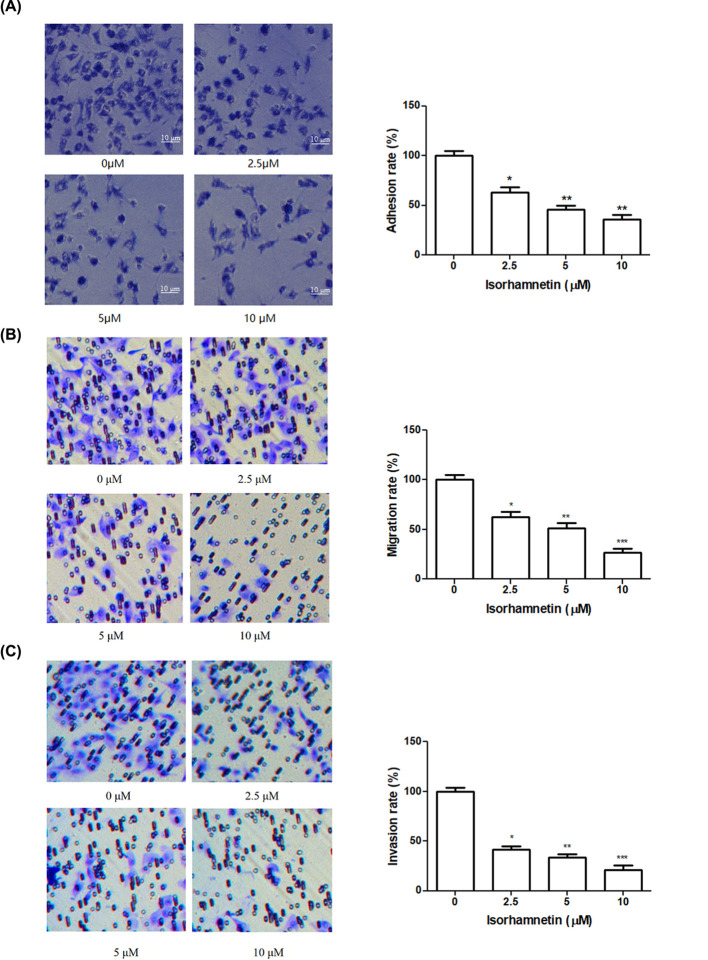
Isorhamnetin inhibits the adhesion, migration, and invasion of A549 cells (**A**) Effects of Isorhamnetin (2.5, 5 and 10 μM) on the adhesion of A549 lung cancer cells. (**B**) Effects of Isorhamnetin (2.5, 5 and 10 μM) on the migration of A549 lung cancer cells. (**C**) Effects of Isorhamnetin (2.5, 5 and 10 μM) on the invasion of A549 lung cancer cells. Bars represent mean ± standard deviation (n = 3). *P<0.05, **P<0.01, and ***P<0.001 were considered to indicate a statistically significant difference compared with the control.

**Figure 5:**
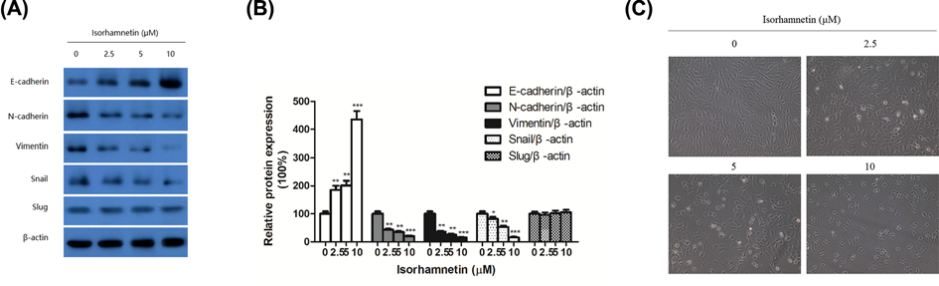
Isorhamnetin inhibits the activation of MMP-2 and MMP-9 in A549 cells (**A**) Effects of Isorhamnetin (0, 2.5, 5, and 10 μM) on the protein expression of E-cadherin, N-cadherin, vimentin, snail, and slug of A549 lung cancer cells. (**B**) Quantitative result of E-cadherin, N-cadherin, vimentin, snail, and slug proteins expression of A549 lung cancer cells treated with Isorhamnetin (0, 2.5, 5, and 10 μM). (**C**) Effects of Isorhamnetin (2.5, 5, and 10 μM) on the cellular morphology of A549 lung cancer cells. Bars represent mean ± S.D. (*n* = 3). *P<0.05, **P<0.01, and ***P<0.001 were considered to indicate a statistically significant difference compared with the control.

